# Real-world outcomes of Finerenone in patients with diabetic kidney disease in Saudi Arabia

**DOI:** 10.1371/journal.pone.0352861

**Published:** 2026-07-01

**Authors:** Mohamed A. Albekery, Ibrahim S. Alhomoud, Khalid A. Alamer, Ahmed A. Alanazi, Abdulmohsen E. Al Mulhem, Ahmed E. Alnaim, Fahad M. Almulhim, Abdulrahman S. Almulhim, Saleh A. Al Makhaytah, Reema H. Aldawsari, Muthana Al Sahlawi, Mohammed Y. Almulhim

**Affiliations:** 1 Department of Pharmacy Practice, College of Clinical Pharmacy, King Faisal University, Al-Ahsa, Saudi Arabia; 2 Department of Pharmacy Practice, College of Pharmacy, Qassim University, Qassim, Saudi Arabia; 3 Department of Pharmacy Practice, College of Pharmacy, Imam Abdulrahman Bin Faisal University, Dammam, Saudi Arabia; 4 Department of Internal Medicine, College of Medicine, King Faisal University, Al-Ahsa, Saudi Arabia; University of Toronto Temerty Faculty of Medicine, CANADA

## Abstract

**Background:**

Diabetic kidney disease (DKD) represents a significant microvascular complication associated with type 2 diabetes mellitus (T2DM), markedly elevating the risk of kidney failure, cardiovascular events, and premature mortality. Despite advancements in therapeutic management, such as renin–angiotensin–aldosterone system (RAAS) blockade and sodium–glucose cotransporter 2 (SGLT2) inhibitors, residual risk remains significant. Finerenone, a novel nonsteroidal and selective mineralocorticoid receptor antagonist (MRA), has demonstrated substantial cardiorenal benefits in clinical trials; however, real-world data, especially from Saudi Arabia, remain limited.

**Method:**

This single-center, retrospective cohort study. All adult patients (≥18 years) who received finerenone as part of routine clinical care were eligible if they met either of the following criteria: (1) documented diabetic kidney disease (DKD) based on KDIGO-aligned clinical criteria, including diabetes mellitus with chronic kidney disease manifested by albuminuria/proteinuria and/or reduced eGFR; (2) and/or a urine protein-to-creatinine ratio (uPCR) >0.3 mg/mg. Longitudinal changes in uPCR, eGFR, and serum potassium were analyzed using linear mixed-effects models adjusted for relevant clinical covariates.

**Results:**

A total of 75 patients prescribed finerenone were screened, of whom 67 met the inclusion criteria. The median age was 63 years, and 53.7% were female. Comorbidities were highly prevalent, including diabetes mellitus (89.6%), hypertension (97.0%), dyslipidemia (92.5%), heart failure (56.8%), and coronary artery disease (40.3%). A significant effect of time on uPCR was observed (F[3,193] = 3.457; P = 0.018), with a mean reduction of 0.464 mg/mg after six months (95% CI, −0.895 to −0.034; P = 0.027). The estimated eGFR slope after finerenone initiation was −1.08 mL/min/1.73 m² per month (95% CI −1.74 to −0.41; P = 0.002), corresponding to an annualized decline of approximately −12.9 mL/min/1.73 m² per year. Serum potassium increased modestly at early follow-up points (F[5,245] = 4.008; P = 0.002), rising by +0.209, + 0.256, and +0.286 mmol/L at the first three readings (P = 0.029, 0.005, and 0.006, respectively), then plateaued thereafter (P > 0.5).

**Conclusion:**

Finerenone use among DKD patients in Saudi Arabia was associated with significant reductions in proteinuria, an early decline in eGFR that requires cautious interpretation, and an overall favorable safety profile. These real-world findings align with results from pivotal clinical trials and support the incorporation of finerenone into standard DKD management. Future multicenter prospective studies are warranted to confirm these outcomes and evaluate long-term cardiorenal benefits.

## Introduction

Diabetes mellitus remains a significant global health challenge, with its prevalence steadily increasing despite considerable progress in both preventive strategies and therapeutic interventions [1]. Currently, the disease affects over half a billion individuals worldwide, and the global burden is projected to escalate sharply to affect an estimated 1.3 billion people by 2050 [2,3]. According to the Global Burden of Disease (GBD) Study, the global age-standardized prevalence of diabetes in 2021 was 6.1%, whereas in Saudi Arabia it reached 18.3% [2]. This represents nearly a threefold increase compared to the global average and reflects a substantial national health burden. Moreover, a recent case-control study conducted in Saudi Arabia involving over 46,000 individuals found that the risk of prediabetes increased by 6.7% for each additional year of age (adjusted odds ratio [aOR]: 1.067; 95% CI: 1.065–1.069) [4]. This growing burden of diabetes and prediabetes underscores the urgent need for targeted prevention and treatment strategies.

Among the chronic complications of diabetes, kidney disease remains one of the most prevalent and clinically significant conditions. Diabetic kidney disease (DKD) affects approximately 30–40 percent of individuals with type 2 diabetes mellitus [5]. It represents a major microvascular complication that substantially increases the risk of kidney failure, atherosclerotic cardiovascular disease, heart failure, and premature death [5–8]. Its burden extends beyond kidney impairment to affect healthcare expenditures among individuals with type 2 diabetes [9,10]. A recent study conducted in Saudi Arabia that included 301 individuals with type 2 diabetes reported a prevalence of 36.8% for a urine albumin-to-creatinine ratio of 30–300 mg/g [11]. This highlights the significant burden of DKD within the region.

Traditional management strategies for DKD focus on optimizing glycemic and blood pressure control, with guideline-recommended use of renin–angiotensin-aldosterone system (RAAS) inhibitors and sodium-glucose cotransporter 2 (SGLT2) inhibitors as first-line therapies [12,13]. In addition, glucagon-like peptide-1 (GLP-1) receptor agonists with demonstrated cardiovascular and kidney benefits should be considered as adjunctive therapy, particularly for individuals with established or high risk of developing atherosclerotic cardiovascular disease (ASCVD) [5,13–15].

Mineralocorticoid receptor overactivation has been identified as a key driver of inflammation and fibrosis in DKD [16,17]. Finerenone, a nonsteroidal and highly selective mineralocorticoid receptor antagonist (MRA), was developed to target this pathogenic pathway [12,18]. Although finerenone does not appear to affect glycemic control, it has been shown to reduce albuminuria and lower the incidence of clinically relevant kidney and cardiovascular events in large randomized controlled trials and meta-analyses [19–23]. These findings broaden the pharmacologic options available for the management of DKD. However, data on the effectiveness and safety of finerenone in real-world clinical settings remain limited. Therefore, this retrospective study was conducted to evaluate its use, effectiveness, and safety in routine clinical practice.

## Methods

### Study design

This single-center retrospective cohort study investigated the effectiveness and safety of finerenone among individuals with DKD receiving routine care between January 1, 2023, and December 31, 2024.

### Ethical approval and participant consent

The research received approval from the Institutional Review Board at Almoosa Health Group in Al-Ahsa, Saudi Arabia, on November 6^th^, 2024 (approval no. ARC-24.11.05).

### Study population

All adult patients (≥18 years) who received finerenone as part of routine clinical care were eligible if they met either of the following criteria: (1) documented diabetic kidney disease (DKD) based on KDIGO-aligned clinical criteria, including diabetes mellitus with chronic kidney disease manifested by albuminuria/proteinuria and/or reduced eGFR; (2) and/or a urine protein-to-creatinine ratio (uPCR) >0.3 mg/mg. Patients were excluded if they had a prior history of kidney transplantation, were expected to receive a transplant within 12 months of therapy initiation, patients on dialysis, had a serum potassium level ≥4.9 mmol/L at the time of finerenone initiation, had uncontrolled glycated hemoglobin (HbA1c) ≥12%, or had uncontrolled systolic blood pressure (SBP) ≥170 mmHg. Additionally, patients with incomplete pre-treatment variables (age, sex, serum creatinine (Scr), eGFR, or serum potassium) were excluded. Kidney biopsy confirmation was not required for DKD classification.

### Study setting

The study was conducted at Almoosa Health Group, a tertiary medical center in Al-Ahsa, Saudi Arabia.

### Data collection

Data were collected from the electronic medical records of eligible patients by screening for various factors, including demographic information, comorbidities, and concurrent medications. Additionally, pre-treatment laboratory values included eGFR, systolic blood pressure, uPCR, potassium, Scr, and HbA1c. Follow-up measurements of uPCR, eGFR, Scr, and potassium were collected at the prespecified intervals for up to 12 months after initiation of finerenone.

Baseline characteristics were collected as the most recent available values recorded in the electronic medical record prior to finerenone initiation. The index date was defined as the date of the first documented prescription of finerenone. All follow-up measurements were referenced relative to this index date. Comorbidities, including heart failure, cardiovascular disease, hypertension, and other baseline conditions, were identified from electronic health records using the corresponding International Classification of Diseases, Tenth Revision (ICD-10) diagnostic codes. Data collection was conducted from January 30, 2025, to April 30, 2025. All data were collected and managed using Microsoft Excel with secure access. Participant confidentiality was maintained through unique, anonymous serial numbers and limited access to data by the investigators only. Informed consent was waived in accordance with the policies of the governmental and local research centers.

### Outcomes

The primary endpoint was to evaluate the efficacy of finerenone, specifically the change in proteinuria, assessed by the uPCR from pre-treatment period and at subsequent 3-month intervals following treatment initiation. Additionally, changes in kidney function were assessed through eGFR slope and Scr levels at baseline, 2 weeks, monthly, and subsequently at 3-month intervals.

Laboratory measurements, including serum creatinine and proteinuria (uPCR), were collected at baseline and during follow-up after initiation of finerenone.

The secondary endpoint was to evaluate the safety profile of finerenone, through monitoring serum potassium concentrations at baseline, 2 weeks, monthly, and subsequently at 3-month intervals, as well as the incidence of all-cause mortality.

### Study analysis

Statistical analyses were performed in IBM SPSS Statistics v26. Continuous variables were summarized as means ± standard deviations when normally distributed or as medians (interquartile range) when skewed, and categorical data were expressed as frequencies and percentages. Baseline predictors of post-treatment hospitalization were first examined with binary logistic regression: each candidate variable underwent univariate screening, and those with *P* < 0.05 were entered into a forward stepwise multivariable model; adjusted odds ratios with 95% confidence intervals are reported, and variance-inflation factors verified that multicollinearity was negligible (all VIF < 2.0). Longitudinal changes in uPCR, serum creatinine, and serum potassium were evaluated using linear mixed-effects models with visit treated as a categorical time variable. To evaluate kidney function trajectory, eGFR was analyzed using a linear mixed-effects model with time treated as a continuous variable based on the prespecified follow-up schedule (baseline, 2 weeks, 1 month, and every 3 months thereafter). All models included a patient-specific random intercept to account for within-participant correlation and were adjusted for clinically relevant covariates (age for all models; baseline eGFR and uPCR for the uPCR model; diabetes and hypertension for the serum creatinine model; and baseline potassium, RAAS inhibitor use, and potassium-binder use for the potassium model). Model parameters were estimated by restricted maximum likelihood, preserving partially observed cases under the missing-at-random assumption. Global Type III F-tests assessed the contribution of time and covariates. Throughout, two-sided p-values < 0.05 were considered statistically significant.

To evaluate kidney function trajectory, eGFR was analyzed using a linear mixed-effects model with follow-up time treated as a continuous variable.

## Results

A total of 75 patients who were prescribed finerenone were initially identified and screened for eligibility. After applying the predefined inclusion criteria, 67 patients were included in the final analysis.

### Baseline characteristics

At baseline, the median age of the study population was 63 years (53.7% female). The prevalence rates of diabetes mellitus, hypertension, and dyslipidemia were 89.6%, 97.0%, and 92.5%, respectively. Cardiovascular disease was also frequent, with 56.8% of patients diagnosed with heart failure and 40.3% with coronary artery disease. Additionally, most of the participants were either overweight or obese (94%), reflecting the cardiometabolic burden typical of DKD. Regarding concurrent medication usage, the majority of individuals were prescribed SGLT2 inhibitors (92.5%) and renin-angiotensin system inhibitors (89.6%). Furthermore, 71.6% of patients utilized incretin-based therapies, 67.2% were prescribed calcium-channel blockers, and 13.4% potassium binders. Table 1 summarizes the baseline characteristics.

**Table 1:** Baseline characteristics (N = 67).

Variable	Total (N = 67)
Age — years (median [IQR])	63 (11)
Female sex — no. (%)	31 (53.7)
Height — cm (median [IQR])	163 (13)
Weight — kg (median [IQR])	84 (19)
Body-mass index — kg/m², n (%)	
18.5–24.9	4 (6)
25–29.9	20 (30)
30–34.9	22 (33)
35–39.9	12 (18)
≥ 40	9 (13.5)
Comorbidities — no. (%)	
DM	60 (90)
HTN	65 (97)
Dyslipidemia	62 (92.5)
CAD	27 (40.5)
PVD	10 (15)
Atrial fibrillation	6 (9)
Stroke	10 (15)
Heart-failure phenotype — no. (%)	
HFpEF	33 (50)
HFmrEF	3 (4.5)
HFrEF	2 (3)
Laboratory data	
HbA1c — % (median [IQR])	7.9 (3)
SBP — mm Hg (mean ± SD)	133.0 ± 15.2
eGFR — mL/min/1.73 m² (mean ± SD)	63.7 ± 24.2
uPCR — mg/mg (mean ± SD)	1.64 ± 1.83
Missing — no. (%)	3 (4.5)
Medication use — no. (%)	
ACEi/ ARB/ ARNI	60 (89.5)
Calcium-channel blocker	45 (67)
Beta-blocker	32 (48)
Diuretics	24 (36)
Spironolactone	1 (1.5)
SGLT2 inhibitors	62 (92.5)
Incretin Therapy	48 (71.5)
Metformin	38 (56.5)
Insulin	26 (39)
Potassium Binders	9 (13.5)
Statins	57 (85)
Finerenone 10 mg	65 (97)
Finerenone 20 mg	2 (3)

Data are presented as mean ± SD, median (IQR), or number (%), as appropriate. Abbreviations: ACEi = angiotensin-converting enzyme inhibitor; ARB = angiotensin II receptor blocker; ARNI = angiotensin receptor–neprilysin inhibitor; BMI = body-mass index; CAD = coronary artery disease; DM = diabetes mellitus; eGFR = estimated glomerular filtration rate; HbA1c = glycated hemoglobin; HFpEF = heart failure with preserved ejection fraction; HFmrEF = heart failure with mildly reduced ejection fraction; HFrEF = heart failure with reduced ejection fraction; HTN = hypertension; IQR = interquartile range; PVD = peripheral vascular disease; SBP = systolic blood pressure; SGLT2 = sodium–glucose cotransporter 2; SD = standard deviation; uPCR = Urine Protein-to-Creatinine Ratio.

### Proteinuria

**Fig 1:**
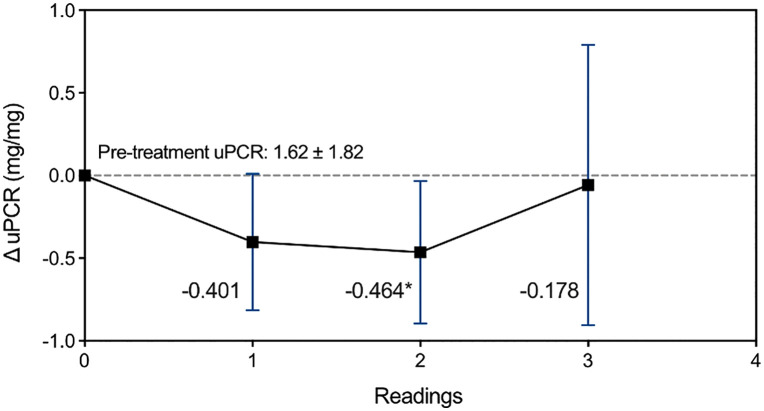
Changes in urine protein-to-creatinine ratio (uPCR) over time following finerenone initiation. Estimated marginal means of uPCR (mg/mg) derived from the linear mixed-effects model are plotted at baseline and at 3, 6, and 9 months post-treatment. A statistically significant reduction in uPCR was observed at 6 months compared to baseline (mean difference −0.464 mg/mg; 95% CI, −0.895 to −0.034; P = 0.027). Error bars represent 95% confidence intervals. Abbreviation: uPCR, urine protein-to-creatinine ratio.

A linear mixed model (LMM) showed a significant effect of time on uPCR levels (F[3,193] = 3.457, *P* = 0.018), indicating that uPCR values varied across follow-up visits (Table 2). After 6 months, uPCR decreased by 0.464 mg/mg from baseline (95% CI, −0.895, −0.034; *P* = 0.027), whereas changes at other time points were not statistically significant as presented in Table 3 and Fig 1.

**Table 2:** uPCR Score across different time points after adjusting for covariates.

Source	F-Statistic	P-Value
Intercept	2.611	0.108
Time	3.457	0.018
Age	0.845	0.359
Pre-treatment eGFR	0.444	0.506
Pre-treatment uPCR	447.687	<0.001

Dependent Variable: uPCR Result.

Abbreviations: eGFR, estimated glomerular filtration rate; uPCR, Urine protein-to-creatinine ratio.

**Table 3:** Pairwise comparisons of uPCR changes from baseline (estimated marginal means).

Compared to	Post-Treatment Reading	Mean Difference (Δ from Baseline)	Std. Error	df	P-Value	95% CI for Difference
Baseline	3 Months	−0.401	0.155	193	0.063	−0.815 to 0.012
	6 Months	−0.464*	0.161	193	0.027	−0.895 to −0.034
	9 Months	−0.178	0.249	193	0.999	−0.841 to 0.485

*. The mean difference is significant at the.05 level.

^a.^ Dependent Variable: uPCR Result.

^b.^ Adjustment for multiple comparisons: Bonferroni.

^c.^ Abbreviation: uPCR, Urine protein-to-creatinine ratio.

### Estimated glomerular filtration rate (eGFR)

**Fig 2:**
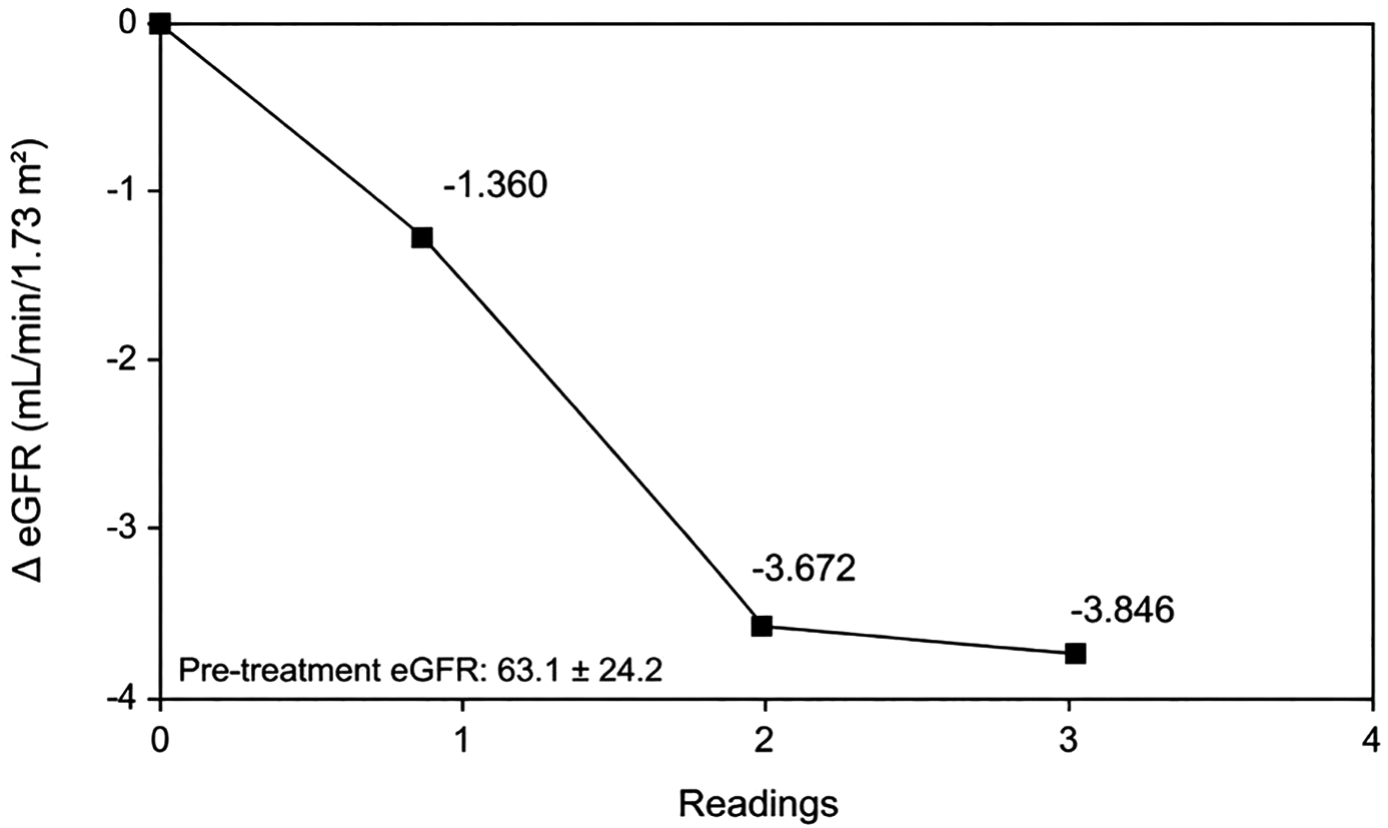
Trajectory of estimated glomerular filtration rate (eGFR) over time following finerenone initiation. Individual patient eGFR trajectories and the overall fitted slope from the linear mixed-effects model are displayed across all prespecified follow-up time points (baseline, 2 weeks, 1 month, and 3-month intervals thereafter). The estimated eGFR slope was −1.08 mL/min/1.73 m² per month (95% CI, −1.74 to −0.41; P = 0.002), corresponding to an annualized decline of approximately −12.9 mL/min/1.73 m² per year. Abbreviation: eGFR, estimated glomerular filtration rate.

To evaluate kidney function trajectory, eGFR slope was analyzed using a linear mixed-effects model with follow-up time treated as a continuous variable. The estimated eGFR slope after finerenone initiation was −1.08 mL/min/1.73 m² per month (95% CI −1.74 to −0.41; P = 0.002), corresponding to an annualized decline of approximately −12.9 mL/min/1.73 m² per year (Table 4). This indicates a statistically significant decline in kidney function over follow-up (Fig 2). Age and baseline uPCR were not significantly associated with eGFR trajectory.

**Table 4:** Linear mixed-effects model estimating eGFR slope.

Variable	β coefficient	Standard error	95% CI	P-value
Time (months)	−1.075	0.339	−1.744 to −0.406	0.002
Age	0.010	0.048	−0.085 to 0.105	0.836
Baseline- PCR	−2.061	1.572	−5.203 to 1.082	0.195

### Potassium level

**Fig 3:**
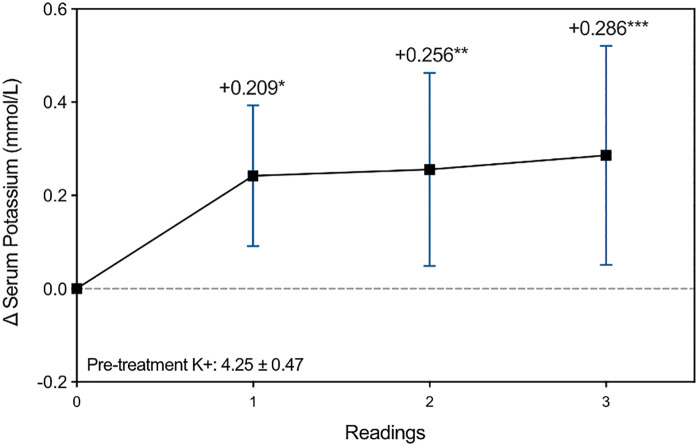
Changes in serum potassium levels over time following finerenone initiation. Estimated marginal means of serum potassium (mmol/L) from the linear mixed-effects model are shown at baseline and at 2 weeks, 1 month, 3 months, and subsequent follow-up readings. Serum potassium increased significantly at the first three post-treatment readings (+0.209 mmol/L at 2 weeks, P = 0.029; + 0.256 mmol/L at 1 month, P = 0.005; + 0.286 mmol/L at 3 months, P = 0.006) and then plateaued thereafter (P > 0.5). Error bars represent 95% confidence intervals. No cases of hospitalization for hyperkalemia were recorded. Abbreviations: K, serum potassium; mmol/L, millimoles per liter.

A LMM demonstrated a significant effect of time on serum potassium levels (F[5,245] = 4.008, *P* = 0.002), indicating that potassium values varied across follow-up points (Table 5). Serum potassium increased significantly from baseline to the 1st, 2nd, and 3rd post-treatment readings (+0.209 mmol/L, + 0.256 mmol/L, and +0.286 mmol/L; *P* = 0.029, 0.005, and 0.006, respectively) as presented in Table 6 and Fig 3. However, no significant changes were observed at the 4th and 5th readings (*P* > 0.5), suggesting stabilization of the potassium level after the 3rd reading.

**Table 5:** Potassium score across different time points adjusting for different covariates.

Source	F-Statistic	P-Value
Intercept	79.289	<0.0001
Time	4.008	0.002
ACEi/ARBs/ARNI	0.346	0.557
Potassium Baseline	100.617	<0.001

Dependent Variable: K Result.

Abbreviations: ACEi/ARBs/ARNI, angiotensin-converting enzyme inhibitors/angiotensin receptor blockers/angiotensin receptor–neprilysin inhibitors.

**Table 6:** Pairwise comparisons of serum potassium changes from baseline (estimated marginal means).

Compared to	Post-Treatment Reading	Mean Difference (Δ from Baseline)	Std. Error	df	P-Value	95% CI for Difference
Baseline	2 Weeks	+0.209*	0.067	245	0.029	0.011 to 0.407
	1 Month	+0.256*	0.070	245	0.005	0.049 to 0.463
	3 Months	+0.286*	0.079	245	0.006	0.051 to 0.521

*. The mean difference is significant at the 0.05 level.

^a.^ Dependent Variable: Serum Potassium Result.

^c.^ Adjustment for multiple comparisons: Bonferroni.

## Discussion

Our findings demonstrated a statistically significant reduction in proteinuria, most apparent between pre-treatment and the second visit, with mean uPCR decreasing from 1.64 ± 1.83 mg/mg (*P* = 0.027). Kidney function showed an early statistically significant decline based on the slope analysis (−1.08 mL/min/1.73 m² per month; 95% CI −1.74 to −0.41; P = 0.002), corresponding to an annualized estimate of approximately −12.9 mL/min/1.73 m² per year; however, this estimate should be interpreted cautiously because it was derived from short follow-up and may not reflect the chronic long-term eGFR slope. In addition, the observational design and limited sample size prevent definitive conclusions about long-term kidney outcomes. While the modest sample size limited the ability to robustly assess clinically meaningful endpoints such as sustained ≥40% or ≥57% eGFR decline, the observed pattern is consistent with mechanistic expectations of non-steroidal MRA.

These results align with pivotal randomized trials. FIDELIO-DKD demonstrated an 18% risk reduction in kidney failure, sustained ≥40% decline in eGFR, or kidney death (HR 0.82; 95% CI, 0.73–0.93; *P* = 0.001), and reduced urine albumin-to-creatinine ratio (uACR) by 31% (least-squares mean ratio 0.69, CI, 0.66–0.71; *P* < 0.001) [19], while in FIGARO-DKD, finerenone reduced end stage kidney disease (ESKD) risk by 36% (HR 0.64; 95% CI: 0.41–0.995; P = 0.046), and reduced uACR by 32% in 4 months (least-squares mean ratio 0.68; 95% CI: 0.65–0.70; P < 0.001). Additionally, it extended benefits to cardiovascular outcomes, showing a 13% reduction in the composite of cardiovascular death, nonfatal myocardial infarction, nonfatal stroke, or hospitalization for heart failure (HR 0.87; 95% CI, 0.76–0.98; *P* = 0.03) [20]. The FIDELITY pooled analysis further confirmed these complementary benefits, reporting a 23% reduction in kidney outcomes and a 14% reduction in cardiovascular events [21]. Unlike these trials, however, our cohort included widespread background use of SGLT2 inhibitors (92.5%), suggesting that finerenone can be safely layered onto contemporary multidrug regimens, a question that has been recently addressed by the CONFIDENCE trial [24]. Combination therapy with finerenone and empagliflozin produced greater reductions in albuminuria than either agent alone, without unexpected safety concerns [24].

Emerging real-world studies further support the reno-protective properties of finerenone within more diverse clinical cohorts. In a recent observational study involving patients with DKD complicated by nephrotic syndrome (n = 9), baseline uPCR was 6.6 ± 2.0; treatment with finerenone was associated with a substantial reduction in proteinuria, as evidenced by a mean uPCR reduction to −0.6 ± 3.9 (P = 0.593) over a mean follow-up period of approximately 10 months. Furthermore, the slope of eGFR decline improved from −3.1 ± 4.9 to −1.7 ± 3.2 mL/min/1.73 m² (P = 0.173), implying a potential stabilization of renal function in individuals exhibiting severe albuminuria [25]. Additionally, another real-world study investigated patients with DKD and advanced CKD, specifically those with a baseline eGFR < 25 mL/min/1.73 m². Within this cohort (n = 9), finerenone therapy demonstrably mitigated the decline in kidney function, as indicated by an improvement in the mean eGFR slope from −7.63 ± 9.84 mL/min/1.73 m²/year prior to treatment to −1.44 ± 3.17 mL/min/1.73 m²/ 6 months after treatment initiation (P = 0.038), without a significant increase in serum potassium levels [26]. In contrast, our cohort demonstrated an estimated eGFR slope decline of −1.08 mL/min/1.73 m² per month, corresponding to approximately −12.9 mL/min/1.73 m² per year. However, the annualized eGFR slope should be interpreted with caution, as it was derived from a relatively short follow-up period and was largely influenced by early post-initiation changes in eGFR. Previous finerenone studies have shown an early decline in eGFR after treatment initiation, followed by stabilization and a slower rate of long-term decline. Therefore, our annualized eGFR slope should not be interpreted as evidence of sustained long-term loss of kidney function. Rather, it is likely to reflect the early dip in eGFR, a short observation window, and limited availability of later eGFR readings. Longer prospective follow-up is needed to determine whether kidney function stabilizes over time. Collectively, these findings extend the evidence beyond the populations typically enrolled in randomized trials and reinforce the growing body of real-world data suggesting that finerenone may provide clinically meaningful renal benefits across a wide spectrum of DKD severity, including patients with nephrotic-range proteinuria or advanced CKD who are often underrepresented in clinical trials. Mechanistically, our findings are consistent with the anti-inflammatory and anti-fibrotic effects of non-steroidal MRA described in translational and clinical reviews [12,16–18], and align with ADA and KDIGO guidance, which endorse finerenone for patients with type 2 diabetes, CKD, and persistent albuminuria despite optimized RAAS blockade [5,13].

For the safety outcomes, no cases of hospitalization for hyperkalemia were recorded, although a statistically significant but clinically modest rise in mean serum potassium from pre-treatment period across follow-up (Δ = +0.209 mmol/L at first visit, *P* = 0.029; Δ = +0.256 mmol/L at second visit, *P* = 0.005; Δ = +0.286 mmol/L at third visit, *P* = 0.006). This contrasts with the pivotal randomized trials, in which hyperkalemia occurred more frequently with finerenone compared with placebo 18.3% versus 9.0% in FIDELIO-DKD and 10.8% versus 5.3% in FIGARO-DKD [19,20]. By comparison, the Japanese FOUNTAIN study, which included 1029 finerenone initiators across two nationwide cohorts, reported hyperkalemia in approximately 2–3%, with no associated hospitalizations [27]. These findings suggest that although finerenone is associated with an increased risk of hyperkalemia, only a modest rise in serum potassium levels was observed in our cohort during follow-up. Importantly, no serious adverse events or hospitalizations related to hyperkalemia were reported throughout the study period. However, these findings should be interpreted cautiously, as the limited sample size and relatively greater use of potassium binders (13.4%) may have contributed to the absence of hyperkalemia events.

A major strength of this study is its real-world design, offering early insights into finerenone use in a Saudi DKD population—a group underrepresented in clinical trials. However, limitations must be acknowledged. The retrospective design, relatively small sample size, single center study, and lack of a control group limit causal inference. Also, we excluded the fourth and fifth readings from the analysis due to extensive missing data, which limited the reliability of these measurements. Furthermore, proteinuria was assessed using the uPCR rather than uACR, which is the preferred marker for evaluating albuminuria and kidney disease progression. uACR measurements were not consistently available because uPCR was the routinely documented measure in our institution during the study period. While uPCR reflects total urinary protein excretion, it does not specifically quantify albuminuria, which may limit direct comparability with studies that use uACR. Additionally, because most patients were receiving both RAS inhibitors and SGLT2 inhibitors, along with finerenone, subgroup comparisons across different background therapy combinations were not feasible and would have yielded unstable estimates due to small sample sizes. Nonetheless, the findings provide valuable insight into current clinical practice, including the prevalence of potassium binder use among this patient population. Considering the high cost of potassium binders, future studies should evaluate the economic and clinical implications of adding these agents to finerenone therapy in clinical settings. Additionally, future studies with larger sample size, more diverse patient populations and longer follow-up durations are warranted to validate these real-world findings on finerenone and provide a more comprehensive understanding of its effectiveness and safety in routine clinical practice.

## Conclusion

In conclusion, this study provides the first real-world evidence on finerenone use among DKD patients in Saudi Arabia. Finerenone was associated with significant reductions in proteinuria, a modest decline in kidney function over the follow-up period, and a good safety profile, with no new safety signals identified in this cohort. These results mirror the efficacy and safety reported in randomized controlled trials and add valuable regional data supporting finerenone’s incorporation into clinical practice. Future prospective, multicenter studies with larger cohorts and longer follow-up are warranted to confirm these findings and further evaluate long-term kidney and cardiovascular outcomes, cost-effectiveness, and strategies to mitigate hyperkalemia in high-risk patients.

## Supporting information

S1 File Supplementary tables: This file contains the following supplementary tables. S1 Table. Baseline Predictors of Post Treatment Multivariable Binary Logistic Regression; S2 Table. PCR Changes Over Time; S3 Table. PCR Changes Over Time Using Pairwise Comparisons; S4 Table. eGFR Changes Over Time; S5 Table. eGFR Changes Over Time Using Pairwise Comparisons; S6 Table. Serum Creatinine Changes Over Time; S7 Table. Serum Creatinine Changes Over Time Using Pairwise Comparisons; S8 Table. Serum Potassium Changes Over Time; and S9 Table. Serum Potassium Changes Over Time Using Pairwise Comparisons.
(PDF)
