# Supplementary material for: Real-world outcomes of Finerenone in patients with diabetic kidney disease in Saudi Arabia
Source: PLoS One. 2026 Jul 1;21(7):e0352861. doi: 10.1371/journal.pone.0352861 (PMC13322498; doi:10.1371/journal.pone.0352861)
Supplement: S1 File — S1 Table. Baseline Predictors of Post Treatment Multivariable Binary Logistic Regression; S2 Table. PCR Changes Over Time; S3 Table. PCR Changes Over Time Using Pairwise Comparisons; S4 Table. eGFR Changes Over Time; S5 Table. eGFR Changes Over Time Using Pairwise Comparisons; S6 Table. Serum Creatinine Changes Over Time; S7 Table. Serum Creatinine Changes Over Time Using Pairwise Comparisons; S8 Table. Serum Potassium Changes Over Time; and S9 Table. Serum Potassium Changes Over Time Using Pairwise Comparisons. (PDF) [file pone.0352861.s001.pdf]

Supplementary Data:

S1 Table. Baseline Predictors of Post-Treatment Hospitalization: Univariate and Multivariable Binary Logistic Regression:

		Univariate Unadjusted LR			Multivariate LR Analysis Multivariate LR (Stepwise FW)		
Variables	Groups	ODDS	95% CI	p	ODDS	95% CI	p
Sex	Male (Ref.)						
	Female	.215	[.071-.649]	.006	.241	[.041-1.419]	.116
Age (years)	(Cont.)	.990	[.942-1.040]	.689	-	-	-
Height (cm)	(Cont.)	.951	[.899-1.007]	.083	-	-	-
Weight (kg)	(Cont.)	1.002	[.971-1.034]	.915	-	-	-
BMI (kg/m²)	18.5-24.9 (Ref.)				-	-	-
	25.0-29.9	.000	[0.000 - 0.000]	0.999	-	-	-
	30.0-34.9	.000	[0.000 - 0.000]	0.999	-	-	-
	35.0-39.9	.000	[0.000 - 0.000]	0.999	-	-	-
	Above 40	.000	[0.000 - 0.000]	0.999	-	-	-
BMI	Cont.	1.048	[.969-1.135]	.241	-	-	-
DM	No (Ref.)				-	-	-
	Yes	0.991	[.216- 4.556]	0.991	-	-	-
HTN	No (Ref.)				-	-	-
	Yes	0.0	[0.000 - 0.000]	0.999	-	-	-
Dyslipidemia	No (Ref.)				-	-	-
	Yes	0.885	[0.137 - 5.693]	0.897	-	-	-
HFpEF	No (Ref.)						
	Yes	6.333	[2.067 - 19.407]	0.001	15.978	[1.747-146.16]	.014
HFmrEF	No (Ref.)				-	-	-

	Yes	0.833	[0.072 - 9.688]	0.884	-	-	-
HFrEF	No (Ref.)				-	-	-
	Yes	0.0	[0.000 - 0.000]	0.999	-	-	-
CAD	No (Ref.)						
	Yes	2.839	[1.018 - 7.917]	0.046	1.766	[.395-7.885]	.457
Peripheral vascular disease	No (Ref.)				-	-	-
	Yes	1.85	[0.478 - 7.163]	0.373	-	-	-
Atrial fibrillation	No (Ref.)				-	-	-
	Yes	0.826	[0.140 - 4.872]	0.833	-	-	-
Stroke	No (Ref.)				-	-	-
	Yes	0.37	[0.072 - 1.900]	0.233	-	-	-
HbA1c	(Cont.)	1.01	[0.927 - 1.101]	0.814	-	-	-
SBP	(Cont.)	0.991	[0.958 - 1.024]	0.579	-	-	-
eGFR (mL/min/1.73 m²)	<25 (Ref.)				-	-	-
	25 to <45	3.000	[.220-40.931]	.410	-	-	-
	45 to <60	1.200	[.089-16.239]	.891	-	-	-
	> 60	.750	[.060-9.319]	.823	-	-	-
PCR (mg/mg)	<3				-	-	-
	3 to <30	1.7	[0.438 - 6.604]	0.443	-	-	-
ACEi/ARBs / ARNI	No (Ref.)				-	-	-
	Yes	0.772	[0.158 - 3.772]	0.749	-	-	-
Calcium-channel blockers	No (Ref.)				-	-	-
	Yes	0.452	[0.158 - 1.287]	0.137	-	-	-
Beta-blockers	No (Ref.)				-	-	-
	Yes	1.697	[0.625 - 4.606]	0.299	-	-	-
Diuretics	No (Ref.)				-	-	-
	Yes	1.012	[0.361 - 2.842]	0.981	-	-	-
	No (Ref.)				-	-	-

Spironolactone	Yes	1.0	[0.000 - 0.000]	1.00	-	-	-
SGLT2 inhibitors	No (Ref.)				-	-	-
	Yes	0.885	[0.137 - 5.693]	0.897	-	-	-
Incretin Therapy	No (Ref.)				-	-	-
	Yes	2.184	[0.531 - 8.981]	0.279	-	-	-
Metformin	No (Ref.)				-	-	-
	Yes	0.737	[0.272 - 1.998]	0.548	-	-	-
Insulin	No (Ref.)				-	-	-
	Yes	2.143	[0.739 - 6.211]	0.160	-	-	-
Potassium Binders	No (Ref.)						
	Yes	8.400	[1.561 - 45.204]	0.013	23.27	[1.977-274.108]	.012
Statins	No (Ref.)				-	-	-
	Yes	0.676	[0.163 - 2.803]	0.589	-	-	-
Finerenone	No (Ref.)				-	-	-
	Yes	2949997539.119	[0.000 - 0.000]	0.999	-	-	-

Abbreviations: BMI, body mass index; DM, diabetes mellitus; HTN, hypertension; HFpEF, heart failure with preserved ejection fraction; HFmrEF, heart failure with mildly reduced ejection fraction; HFrEF, heart failure with reduced ejection fraction; CAD, coronary artery disease; HbA1c, glycated hemoglobin; SBP, systolic blood pressure; eGFR, estimated glomerular filtration rate; PCR, protein-to-creatinine ratio; ACEi/ARBs/ARNI, angiotensin-converting enzyme inhibitors/angiotensin receptor blockers/angiotensin receptor–neprilysin inhibitors; SGLT2 inhibitors, sodium–glucose cotransporter-2 inhibitors.

S2 Table. PCR Changes over time:

Descriptive Statistics

PCR_Time	Count	Mean	Standard Deviation	Coefficient of Variation

PCR (Baseline) (Pre-Treatment) Result	PCR Result	65	1.6271	1.82952	112.4%
	Baseline_PCR	65	1.6271	1.82952	112.4%
	Baseline_eGFR	65	63.1077	24.27391	38.5%
	Age	65	61.8308	10.00870	16.2%
PCR 1st reading (post-treatment) Result	PCR Result	64	1.2395	1.48519	119.8%
	Baseline_PCR	64	1.6445	1.83852	111.8%
	Baseline_eGFR	64	63.5469	24.20411	38.1%
	Age	64	61.4531	10.03475	16.3%
PCR 2nd reading (post-treatment) Result	PCR Result	55	1.2622	1.29413	102.5%
	Baseline_PCR	55	1.7625	1.93768	109.9%
	Baseline_eGFR	55	62.2182	24.96864	40.1%
	Age	55	61.1636	9.20299	15.0%
PCR 3rd reading (post-treatment) Result	PCR Result	16	1.9875	2.14461	107.9%
	Baseline_PCR	16	2.4288	2.71408	111.7%
	Baseline_eGFR	16	60.2500	23.87328	39.6%
	Age	16	65.5000	7.58068	11.6%
Total	PCR Result	200	1.4315	1.62267	113.4%
	Baseline_PCR	200	1.7340	1.94061	111.9%
	Baseline_eGFR	200	62.7750	24.24714	38.6%
	Age	200	61.8200	9.62605	15.6%

16

S3 Table. PCR Changes over time using Pairwise Comparisons:

Pairwise Comparisons[a]

(I) PCR_Time	(J) PCR_Time	Mean Difference (I-J)	Std. Error	df	Sig.[c]	95% Confidence Interval for Difference[c] Lower Bound	Upper Bound
PCR (Baseline) (Pre-Treatment) Result	PCR 1st reading (post-treatment) Result	.401	.155	193	.063	-.012	.815
	PCR 2nd reading (post-treatment) Result	.464*	.161	193	.027	.034	.895
	PCR 3rd reading (post-treatment) Result	.178	.249	193	1.000	-.485	.841
PCR 1st reading (post-treatment) Result	PCR (Baseline) (Pre-Treatment) Result	-.401	.155	193	.063	-.815	.012
	PCR 2nd reading (post-treatment) Result	.063	.162	193	1.000	-.369	.495
	PCR 3rd reading (post-treatment) Result	-.223	.249	193	1.000	-.888	.441

PCR 2nd reading (post-treatment) Result	PCR (Baseline) (Pre-Treatment) Result	-.464*	.161	193	.027	-.895	-.034
	PCR 1st reading (post-treatment) Result	-.063	.162	193	1.000	-.495	.369
	PCR 3rd reading (post-treatment) Result	-.287	.253	193	1.000	-.961	.388
PCR 3rd reading (post-treatment) Result	PCR (Baseline) (Pre-Treatment) Result	-.178	.249	193	1.000	-.841	.485
	PCR 1st reading (post-treatment) Result	.223	.249	193	1.000	-.441	.888
	PCR 2nd reading (post-treatment) Result	.287	.253	193	1.000	-.388	.961

Based on estimated marginal means

*. The mean difference is significant at the .05 level.

a. Dependent Variable: PCR Result.

c. Adjustment for multiple comparisons: Bonferroni.

S4 Table. eGFR Changes over time:

Descriptive Statistics

eGFR_Time		Count	Mean	Standard Deviation	Coefficient of Variation
	eGFR Result	65	63.1077	24.27391	38.5%

eGFR (Baseline) (Pre-Treatment) Result	Baseline_eGFR	65	63.1077	24.27391	38.5%
	Baseline_PCR	65	1.6271	1.82952	112.4%
	Age	65	61.8308	10.00870	16.2%
eGFR 1st reading (post-treatment) Result	eGFR Result	65	61.7431	24.61034	39.9%
	Baseline_eGFR	65	63.1077	24.27391	38.5%
	Baseline_PCR	65	1.6271	1.82952	112.4%
	Age	65	61.2000	10.16305	16.6%
eGFR 2nd reading (post-treatment) Result	eGFR Result	60	59.2333	24.04612	40.6%
	Baseline_eGFR	60	62.9500	24.70808	39.3%
	Baseline_PCR	60	1.6973	1.87031	110.2%
	Age	60	61.2333	10.35041	16.9%
eGFR 3rd reading (post-treatment) Result	eGFR Result	43	56.3023	26.75954	47.5%
	Baseline_eGFR	43	59.9070	25.89430	43.2%
	Baseline_PCR	43	1.6388	1.94463	118.7%
	Age	43	60.7209	10.74227	17.7%
eGFR 4th reading (post-treatment) Result	eGFR Result	29	51.7241	24.50204	47.4%
	Baseline_eGFR	29	57.0345	25.74807	45.1%
	Baseline_PCR	29	1.8269	2.29569	125.7%
	Age	29	58.9655	12.10514	20.5%
	eGFR Result	16	50.0000	19.27001	38.5%

eGFR 5th reading (post-treatment) Result	Baseline_eGFR	16	56.1875	19.07955	34.0%
	Baseline_PCR	16	2.3850	2.74697	115.2%
	Age	16	58.7500	13.58676	23.1%
Total	eGFR Result	278	58.9579	24.61072	41.7%
	Baseline_eGFR	278	61.5468	24.42294	39.7%
	Baseline_PCR	278	1.7085	1.95915	114.7%
	Age	278	60.9065	10.63228	17.5%

S5 Table. eGFR Changes over time using Pairwise Comparisons:

Pairwise Comparisons[a]

						95% Confidence Interval for Difference[b]	
(I) eGFR_Time	(J) eGFR_Time	Mean Difference (I-J)	Std. Error	df	Sig.[b]	Lower Bound	Upper Bound
eGFR (Baseline) (Pre-Treatment) Result	eGFR 1st reading (post-treatment) Result	1.360	1657.221	306679414038931	1.000	-4862.914	4865.634
	eGFR 2nd reading (post-treatment) Result	3.672	1657.221	40183847.105	1.000	-4860.028	4867.372

	eGFR 3rd reading (post-treatment) Result	3.846	1657.221	2449190.923	1.000	-4860.433	4868.125
	eGFR 4th reading (post-treatment) Result	5.629	1657.221	510481366.248	1.000	-4858.647	4869.904
	eGFR 5th reading (post-treatment) Result	6.152	1657.222	350131.440	1.000	-4858.159	4870.462
eGFR 1st reading (post-treatment) Result	eGFR (Baseline) (Pre-Treatment) Result	-1.360	1657.221	306679414038931	1.000	-4865.634	4862.914
	eGFR 2nd reading (post-treatment) Result	2.312	1657.221	40183811.013	1.000	-4861.388	4866.012
	eGFR 3rd reading (post-treatment) Result	2.485	1657.221	2449190.050	1.000	-4861.794	4866.765
	eGFR 4th reading (post-treatment) Result	4.268	1657.221	510484629.270	1.000	-4860.007	4868.544
	eGFR 5th reading (post-	4.791	1657.222	350131.338	1.000	-4859.519	4869.102

	treatment) Result						
eGFR 2nd reading (post-treatment) Result	eGFR (Baseline) (Pre-Treatment) Result	-3.672	1657.221	40183847.105	1.000	-4867.372	4860.028
	eGFR 1st reading (post-treatment) Result	-2.312	1657.221	40183811.013	1.000	-4866.012	4861.388
	eGFR 3rd reading (post-treatment) Result	.173	1657.221	38174553.363	1.000	-4863.527	4863.874
	eGFR 4th reading (post-treatment) Result	1.956	1657.221	814245589.761	1.000	-4862.319	4866.232
	eGFR 5th reading (post-treatment) Result	2.479	1657.222	1504550.484	1.000	-4861.806	4866.765
eGFR 3rd reading (post-treatment) Result	eGFR (Baseline) (Pre-Treatment) Result	-3.846	1657.221	2449190.923	1.000	-4868.125	4860.433
	eGFR 1st reading (post-treatment) Result	-2.485	1657.221	2449190.050	1.000	-4866.765	4861.794

	eGFR 2nd reading (post-treatment) Result	-.173	1657.221	38174553.363	1.000	-4863.874	4863.527
	eGFR 4th reading (post-treatment) Result	1.783	1657.222	8294631.645	1.000	-4862.494	4866.060
	eGFR 5th reading (post-treatment) Result	2.306	1657.222	15900252.104	1.000	-4861.973	4866.585
eGFR 4th reading (post-treatment) Result	eGFR (Baseline) (Pre-Treatment) Result	-5.629	1657.221	510481366.248	1.000	-4869.904	4858.647
	eGFR 1st reading (post-treatment) Result	-4.268	1657.221	510484629.270	1.000	-4868.544	4860.007
	eGFR 2nd reading (post-treatment) Result	-1.956	1657.221	814245589.761	1.000	-4866.232	4862.319
	eGFR 3rd reading (post-treatment) Result	-1.783	1657.222	8294631.645	1.000	-4866.060	4862.494
	eGFR 5th reading (post-	.523	1657.223	708776.529	1.000	-4863.772	4864.818

	treatment) Result						
eGFR 5th reading (post-treatment) Result	eGFR (Baseline) (Pre-Treatment) Result	-6.152	1657.222	350131.440	1.000	-4870.462	4858.159
	eGFR 1st reading (post-treatment) Result	-4.791	1657.222	350131.338	1.000	-4869.102	4859.519
	eGFR 2nd reading (post-treatment) Result	-2.479	1657.222	1504550.484	1.000	-4866.765	4861.806
	eGFR 3rd reading (post-treatment) Result	-2.306	1657.222	15900252.104	1.000	-4866.585	4861.973
	eGFR 4th reading (post-treatment) Result	-.523	1657.223	708776.529	1.000	-4864.818	4863.772

Based on estimated marginal means

a. Dependent Variable: eGFR Result.

b. Adjustment for multiple comparisons: Bonferroni.

S6 Table. Serum Creatinine Changes over time:

Descriptive Statistics

Serum Creatinine_Time		Count	Mean	Standard Deviation	Coefficient of Variation
	Scr Result	65	115.2491	49.77864	43.2%

Scr (Baseline) (Pre-Treatment) Result	DMYesNo	65	.8769	.33108	37.8%
	HTNYesNo	65	.9692	.17404	18.0%
	Age	65	61.8308	10.00870	16.2%
	Baseline_eGFR	65	63.1077	24.27391	38.5%
	Baseline_PCR	65	1.6271	1.82952	112.4%
Scr 1st reading (post-treatment) Result	Scr Result	65	120.9555	57.63359	47.6%
	DMYesNo	65	.8615	.34807	40.4%
	HTNYesNo	65	.9692	.17404	18.0%
	Age	65	61.2000	10.16305	16.6%
	Baseline_eGFR	65	63.1077	24.27391	38.5%
	Baseline_PCR	65	1.6271	1.82952	112.4%
Scr 2nd reading (post-treatment) Result	Scr Result	60	123.7433	58.65227	47.4%
	DMYesNo	60	.9000	.30253	33.6%
	HTNYesNo	60	.9667	.18102	18.7%
	Age	60	61.2333	10.35041	16.9%
	Baseline_eGFR	60	62.9500	24.70808	39.3%
	Baseline_PCR	60	1.6973	1.87031	110.2%
Scr 3rd reading (post-treatment) Result	Scr Result	43	131.6140	69.64117	52.9%
	DMYesNo	43	.8372	.37354	44.6%
	HTNYesNo	43	.9535	.21308	22.3%
	Age	43	60.7209	10.74227	17.7%
	Baseline_eGFR	43	59.9070	25.89430	43.2%
	Baseline_PCR	43	1.6388	1.94463	118.7%
Scr 4th reading (post-treatment) Result	Scr Result	29	148.4072	91.64030	61.7%
	DMYesNo	29	.8621	.35093	40.7%
	HTNYesNo	29	.9655	.18570	19.2%
	Age	29	58.9655	12.10514	20.5%

	Baseline_eGFR	29	57.0345	25.74807	45.1%
	Baseline_PCR	29	1.8269	2.29569	125.7%
Scr 5th reading (post-treatment) Result	Scr Result	16	136.7188	51.34773	37.6%
	DMYesNo	16	.8125	.40311	49.6%
	HTNYesNo	16	1.0000	.00000	0.0%
	Age	16	58.7500	13.58676	23.1%
	Baseline_eGFR	16	56.1875	19.07955	34.0%
	Baseline_PCR	16	2.3850	2.74697	115.2%
Total	Scr Result	278	125.6425	62.43303	49.7%
	DMYesNo	278	.8669	.34029	39.3%
	HTNYesNo	278	.9676	.17731	18.3%
	Age	278	60.9065	10.63228	17.5%
	Baseline_eGFR	278	61.5468	24.42294	39.7%
	Baseline_PCR	278	1.7085	1.95915	114.7%

S7 Table. Serum Creatinine Changes over time using Pairwise comparison:

Pairwise Comparisons[a]

(I) Serum Creatinine_Time	(J) Serum Creatinine_Time	Mean Difference (I-J)	Std. Error	df	Sig.[b]	95% Confidence Interval for Difference[b] Lower Bound	Upper Bound
Scr (Baseline) (Pre-Treatment) Result	Scr 1st reading (post-treatment) Result	-5.515	6.689	267.000	1.000	-25.327	14.297
	Scr 2nd reading (post-treatment) Result	-7.367	6.828	267	1.000	-27.592	12.858

	Scr 3rd reading (post-treatment) Result	-9.418	7.508	267	1.000	-31.655	12.819
	Scr 4th reading (post-treatment) Result	-19.282	8.555	267	.375	-44.621	6.058
	Scr 5th reading (post-treatment) Result	-5.480	10.747	267	1.000	-37.312	26.352
Scr 1st reading (post-treatment) Result	Scr (Baseline) (Pre-Treatment) Result	5.515	6.689	267.000	1.000	-14.297	25.327
	Scr 2nd reading (post-treatment) Result	-1.852	6.832	267.000	1.000	-22.088	18.383
	Scr 3rd reading (post-treatment) Result	-3.903	7.502	267	1.000	-26.124	18.318
	Scr 4th reading (post-treatment) Result	-13.767	8.546	267.000	1.000	-39.078	11.545
	Scr 5th reading (post-treatment) Result	.035	10.732	267.000	1.000	-31.752	31.821
Scr 2nd reading (post-treatment) Result	Scr (Baseline) (Pre-Treatment) Result	7.367	6.828	267	1.000	-12.858	27.592
	Scr 1st reading (post-treatment) Result	1.852	6.832	267.000	1.000	-18.383	22.088

	Scr 3rd reading (post-treatment) Result	-2.050	7.635	267	1.000	-24.663	20.562
	Scr 4th reading (post-treatment) Result	-11.914	8.658	267.000	1.000	-37.558	13.730
	Scr 5th reading (post-treatment) Result	1.887	10.836	267	1.000	-30.208	33.982
Scr 3rd reading (post-treatment) Result	Scr (Baseline) (Pre-Treatment) Result	9.418	7.508	267	1.000	-12.819	31.655
	Scr 1st reading (post-treatment) Result	3.903	7.502	267	1.000	-18.318	26.124
	Scr 2nd reading (post-treatment) Result	2.050	7.635	267	1.000	-20.562	24.663
	Scr 4th reading (post-treatment) Result	-9.864	9.175	267	1.000	-37.039	17.312
	Scr 5th reading (post-treatment) Result	3.937	11.242	267	1.000	-29.360	37.235
Scr 4th reading (post-treatment) Result	Scr (Baseline) (Pre-Treatment) Result	19.282	8.555	267	.375	-6.058	44.621
	Scr 1st reading (post-treatment) Result	13.767	8.546	267.000	1.000	-11.545	39.078

	Scr 2nd reading (post-treatment) Result	11.914	8.658	267.000	1.000	-13.730	37.558
	Scr 3rd reading (post-treatment) Result	9.864	9.175	267	1.000	-17.312	37.039
	Scr 5th reading (post-treatment) Result	13.801	11.917	267	1.000	-21.494	49.097
Scr 5th reading (post-treatment) Result	Scr (Baseline) (Pre-Treatment) Result	5.480	10.747	267	1.000	-26.352	37.312
	Scr 1st reading (post-treatment) Result	-.035	10.732	267.000	1.000	-31.821	31.752
	Scr 2nd reading (post-treatment) Result	-1.887	10.836	267	1.000	-33.982	30.208
	Scr 3rd reading (post-treatment) Result	-3.937	11.242	267	1.000	-37.235	29.360
	Scr 4th reading (post-treatment) Result	-13.801	11.917	267	1.000	-49.097	21.494

Based on estimated marginal means

a. Dependent Variable: Scr Result.

b. Adjustment for multiple comparisons: Bonferroni.

S8 Table. Serum Potassium Changes over time:

Descriptive Statistics

Serum Potassium		Count	Mean	Standard Deviation	Coefficient of Variation
Serum Potassium at baseline (pre-treatment)	Serum Potassium Result	65	4.2554	.47763	11.2%
	ACEi/ARBs / ARNI	65	.8923	.31240	35.0%
	K Binders	65	.1385	.34807	251.4%
	K_Baseline	65	4.2554	.47763	11.2%
Serum Potassium 1st reading (post-treatment)	Serum Potassium Result	64	4.4672	.50554	11.3%
	ACEi/ARBs / ARNI	64	.9062	.29378	32.4%
	K Binders	64	.1406	.35038	249.2%
	K_Baseline	64	4.2578	.48101	11.3%
Serum Potassium 2nd reading (post-treatment)	Serum Potassium Result	54	4.5093	.45319	10.1%
	ACEi/ARBs / ARNI	54	.8889	.31722	35.7%
	K Binders	54	.1667	.37618	225.7%
	K_Baseline	54	4.2389	.47001	11.1%
Serum Potassium 3rd reading (post-treatment)	Serum Potassium Result	36	4.5694	.48510	10.6%
	ACEi/ARBs / ARNI	36	.9722	.16667	17.1%
	K Binders	36	.2222	.42164	189.7%
	K_Baseline	36	4.2639	.51445	12.1%
Serum Potassium 4th reading (post-treatment)	Serum Potassium Result	24	4.5208	.47638	10.5%
	ACEi/ARBs / ARNI	24	.9583	.20412	21.3%
	K Binders	24	.2917	.46431	159.2%

	K_Baseline	24	4.3208	.55166	12.8%
Serum Potassium 5th reading (post-treatment)	Serum Potassium Result	11	4.6636	.58013	12.4%
	ACEi/ARBs / ARNI	11	.9091	.30151	33.2%
	K Binders	11	.4545	.52223	114.9%
	K_Baseline	11	4.4455	.39589	8.9%
Total	Serum Potassium Result	254	4.4500	.49589	11.1%
	ACEi/ARBs / ARNI	254	.9134	.28182	30.9%
	K Binders	254	.1850	.38910	210.3%
	K_Baseline	254	4.2681	.48369	11.3%

S9 Table. Serum Potassium Changes over time using Pairwise comparisons:

Pairwise Comparisons[a]

(I) Serum Potassium	(J) Serum Potassium	Mean Difference (I-J)	Std. Error	df	Sig.[c]	95% Confidence Interval for Difference[c] Lower Bound	Upper Bound
Serum Potassium at baseline (pre-treatment)	Serum Potassium 1st reading (post-treatment)	-.209*	.067	245.000	.029	-.407	-.011
	Serum Potassium 2nd reading (post-treatment)	-.256*	.070	245	.005	-.463	-.049

	Serum Potassium 3rd reading (post-treatment)	-.286*	.079	245	.006	-.521	-.051
	Serum Potassium 4th reading (post-treatment)	-.191	.091	245	.560	-.462	.080
	Serum Potassium 5th reading (post-treatment)	-.232	.125	245.000	.984	-.603	.140
Serum Potassium 1st reading (post-treatment)	Serum Potassium at baseline (pre-treatment)	.209*	.067	245.000	.029	.011	.407
	Serum Potassium 2nd reading (post-treatment)	-.047	.070	245	1.000	-.255	.161
	Serum Potassium 3rd reading (post-treatment)	-.077	.079	245	1.000	-.312	.159
	Serum Potassium 4th reading (post-treatment)	.018	.091	245	1.000	-.253	.289
	Serum Potassium 5th	-.022	.125	245.000	1.000	-.394	.349

	reading (post-treatment)						
Serum Potassium 2nd reading (post-treatment)	Serum Potassium at baseline (pre-treatment)	.256*	.070	245	.005	.049	.463
	Serum Potassium 1st reading (post-treatment)	.047	.070	245	1.000	-.161	.255
	Serum Potassium 3rd reading (post-treatment)	-.030	.082	245	1.000	-.273	.214
	Serum Potassium 4th reading (post-treatment)	.065	.094	245	1.000	-.213	.343
	Serum Potassium 5th reading (post-treatment)	.024	.127	245.000	1.000	-.352	.401
Serum Potassium 3rd reading (post-treatment)	Serum Potassium at baseline (pre-treatment)	.286*	.079	245	.006	.051	.521
	Serum Potassium 1st reading (post-treatment)	.077	.079	245	1.000	-.159	.312

	Serum Potassium 2nd reading (post-treatment)	.030	.082	245	1.000	-.214	.273
	Serum Potassium 4th reading (post-treatment)	.095	.100	245	1.000	-.202	.391
	Serum Potassium 5th reading (post-treatment)	.054	.132	245	1.000	-.336	.444
Serum Potassium 4th reading (post-treatment)	Serum Potassium at baseline (pre-treatment)	.191	.091	245	.560	-.080	.462
	Serum Potassium 1st reading (post-treatment)	-.018	.091	245	1.000	-.289	.253
	Serum Potassium 2nd reading (post-treatment)	-.065	.094	245	1.000	-.343	.213
	Serum Potassium 3rd reading (post-treatment)	-.095	.100	245	1.000	-.391	.202
	Serum Potassium 5th	-.041	.139	245	1.000	-.451	.370

	reading (post-treatment)						
Serum Potassium 5th reading (post-treatment)	Serum Potassium at baseline (pre-treatment)	.232	.125	245.000	.984	-.140	.603
	Serum Potassium 1st reading (post-treatment)	.022	.125	245.000	1.000	-.349	.394
	Serum Potassium 2nd reading (post-treatment)	-.024	.127	245.000	1.000	-.401	.352
	Serum Potassium 3rd reading (post-treatment)	-.054	.132	245	1.000	-.444	.336
	Serum Potassium 4th reading (post-treatment)	.041	.139	245	1.000	-.370	.451

Based on estimated marginal means

*. The mean difference is significant at the .05 level.

a. Dependent Variable: Serum Potassium Result.

c. Adjustment for multiple comparisons: Bonferroni.
