# Supplementary material for: Real-world outcomes of Finerenone in patients with diabetic kidney disease in Saudi Arabia
Source: PLoS One. 2026 Jul 1;21(7):e0352861. doi: 10.1371/journal.pone.0352861 (PMC13322498; doi:10.1371/journal.pone.0352861)
Supplement: Attachment — Submitted filename: Response to Reviewers.pdf [file pone.0352861.s002.pdf]

Reviewer #1: The authors studied real-world data of finerenone in DKD. The topic is very intriguing and important. These are my comments.

Response:

We sincerely thank the reviewer for their time, thoughtful review of our manuscript, and constructive comments. We truly appreciate their positive feedback and acknowledgment of the importance of our study.

Comment 1: Real-world data on finerenone in DKD is highly important. For changes in GFR, please evaluate using the eGFR slope.

Response 1:

We sincerely thank the reviewer for this valuable and constructive suggestion. In response, we reanalyzed kidney function using a linear mixed-effects model with follow-up time as a continuous variable to estimate the eGFR slope after finerenone initiation, rather than evaluating differences across categorical visits. The revised analysis showed an estimated eGFR slope of −1.08 mL/min/1.73 m^2 per month (95% CI −1.74 to −0.41; P = 0.002), corresponding to an annualized decline of approximately −12.9 mL/min/1.73 m^2 per year. The Methods, Results, and Table 4 have been updated accordingly, and the previous pairwise comparison analysis for eGFR has been removed. Figure B has been retained to visually illustrate the trajectory of eGFR over follow-up.

Comment 2: Please show the changes in urinary protein in the three groups: RASi alone, SGLT2i + RASi, and SGLT2i + RASi + MRA, in accordance with the CONFIDENCE trial.

Response 2:

We thank the reviewer for this important suggestion. Stratification by background kidney-protective therapies is indeed clinically relevant. However, the present study was designed as a real-world evaluation of patients who had already initiated finerenone as part of routine clinical care. Consequently, all patients in the cohort received an MRA (finerenone), and background use of RAS inhibitors and SGLT2 inhibitors was highly prevalent. This resulted in very small and highly imbalanced subgroups for alternative treatment patterns, limiting the validity of comparative analyses across therapy combinations. Because the study was not designed or powered to evaluate treatment combinations and such subgroup analyses would be prone to instability and confounding by indication, we did not perform comparisons analogous to those conducted in randomized trials such as CONFIDENCE. We have clarified this limitation in the revised Discussion.

Comment 3: Please cite and discuss the following paper as real-world data for finerenone.

Effect of finerenone on nephrotic syndrome in patients with diabetic kidney disease.
Metabol Open. 2024 Jun 5;22:100294.

Effect of finerenone on diabetic kidney disease outcomes with estimated glomerular filtration rate below 25 mL/min/1.73 m(2).Metabol Open. 2023 Jul 8;19:100251.

Response 3:

Thank you so much for bringing this up. Both studies have now been cited and discussed in the discussion section.

Reviewer #2: Thank you for allowing me to review your submission. The concept of this paper is good and with some revisions this would be suitable for publication.

Response:

We are grateful to the reviewer for taking the time to evaluate our manuscript and provide insightful feedback. We sincerely appreciate his constructive comments and kind recognition of the significance of our work.

Comment 1:

Main concern is that there needs to justification for using UPCR and not UACR as albuminuria is the accepted marker for kidney disease progression. If data for ACR is available that should be presented, if it is not available then it is important to explain why and what the limitations of using UPCR are.

Response 1:

We thank the reviewer for pointing this out. Urine albumin-to-creatinine ratio (uACR) measurements were not available for all patients, as the urine protein-to-creatinine ratio (uPCR) was the routinely documented measure in our institution (Almoosa Health Group) during the study period. For this reason, uPCR was used as the primary marker of proteinuria in our analysis.

While the use of uPCR instead of uACR had already been acknowledged as a limitation in the discussion section, we have now expanded this explanation to further clarify the rationale for using uPCR and to

explicitly recognize the limitations of relying on uPCR when evaluating albuminuria-related kidney disease progression.

Comment 2:

Baseline albuminuria should also be presented in table 1 and in this cohorts case, the relatively mild degree of baseline proteinuria should be explained in the discussion.

Response 2:

We thank the reviewer for this important insight. Regarding Baseline albuminuria, our cohort reported proteinuria as the protein-to-creatinine ratio (PCR) in mg/mg. According to KDIGO classification, severely increased proteinuria (A3) corresponds to PCR >500 mg/g, which is equivalent to >0.5 mg/mg. The mean baseline value in our cohort was 1.64 mg/mg (~1640 mg/g), clearly within the severely increased range, rather than representing mild proteinuria (1). (Please see the attached table below).

1. Kidney Disease: Improving Global Outcomes (KDIGO) Glomerular Diseases Work Group. KDIGO 2021 Clinical Practice Guideline for the Management of Glomerular Diseases. Kidney International. 2021;100(4Suppl):S1-S276.doi:10.1016/j.kint.2021.05.021.

RELATIONSHIP AMONG CATEGORIES FOR ALBUMINURIA AND PROTEINURIA

	Categories		
**Measure**	**Normal to mildly increased (A1)**	**Moderately increased (A2)**	**Severely increased (A3)**
AER (mg/d)	<30	30–300	>300
PER (mg/d)	<150	150–500	>500
ACR			
(mg/mmol)	<3	3–30	>30
(mg/g)	<30	30–300	>300
PCR			
(mg/mmol)	<15	15–50	>50
(mg/g)	<150	150–500	>500
Protein reagent strip	Negative to trace	Trace to +	+ or greater

Relationships among measurement methods within a category are not exact. For example, the relationships between AER and ACR and between PER and PCR are based on the assumption that average creatinine excretion rate is approximately 1.0 g/d or 10 mmol/d. The conversions are rounded for pragmatic reasons. (For an exact conversion from mg/g of creatinine to mg/mmol of creatinine, multiply by 0.113.) Creatinine excretion varies with age, sex, race and diet; therefore, the relationship among these categories is approximate only. The relationship between urine reagent strip results and other measures depends on urine concentration. ACR, albumin-to-creatinine ratio; AER, albumin excretion rate; PCR, protein-creatinine ratio; PER, protein excretion rate.

Comment 3:

Line 70, delete extra period

Line 108 – “change among DKD individuals” to “among individuals with DKD”

Response 3:

We thank the reviewer for the important suggestion. We have edited accordingly.

Comment 4:

Data collection- Please clarify how baseline characteristics were defined and for example how baseline eGFR was determined (for example was it the most recent value prior to finerenone initiation? Also, please clarify index date determination and chart review procedure. How were endpoints determined?

Response 4:

We thank the reviewer for this important comment.

Baseline characteristics included clinical and laboratory variables measured immediately before starting finerenone, and were obtained from the most recent values. The index date was defined as the date of the first recorded finerenone prescription or administration, and all follow-up assessments were referenced relative to this date. We have updated the data collection section accordingly.

Regarding endpoint determination, the primary effectiveness endpoint was the change in proteinuria, measured by the urine protein-to-creatinine ratio (uPCR) from baseline to follow-up visits. Secondary effectiveness endpoints included changes in kidney function (eGFR and serum creatinine). Safety endpoints included changes in serum potassium. The follow-up schedule and duration were guided by prior RCTs, including the FIGARO-DKD and FIDELIO-DKD studies, to align with established clinical monitoring practices for finerenone therapy.

Comment 5:

Table 2 – instead of label 1st reading, 2nd reading, 3rd reading post treatment change to indicate how many days post, eg. 2 weeks, 1 month, 2 months

Figure B – should say "pre-treatment eGFR

Response 5:

We appreciate the reviewer's observation. The figure has been corrected to indicate "pre-treatment eGFR". In addition, the labels have been revised to reflect the specific follow-up time points. These changes have been applied to the relevant tables (Tables 3 and 6).

Comment 6:

Line 297 – 298, Is data available for serious adverse events or hospitalizations related hyperkalemia after initiation of finerenone? If so this should be presented as well.

Response 6:

We thank the reviewer for their insightful comment. There were no serious adverse events or hospitalizations related to hyperkalemia observed in our cohort during follow-up. We have updated the discussion accordingly (Line 301-304).
