# Supplementary material for: Real-world outcomes of Finerenone in patients with diabetic kidney disease in Saudi Arabia
Source: PLoS One. 2026 Jul 1;21(7):e0352861. doi: 10.1371/journal.pone.0352861 (PMC13322498; doi:10.1371/journal.pone.0352861)
Supplement: Attachment — Submitted filename: Response to Reviewers.docx [file pone.0352861.s003.docx]

**Editor:** Thank you to the authors for their revisions and responses to the reviewer's comments. There are a few outstanding elements of the paper that warrant further attention.

**Response:**

We sincerely thank the editor for his thoughtful comments and for acknowledging our revisions and responses. We appreciate the additional feedback provided and have carefully addressed all outstanding points raised. The corresponding revisions have been incorporated into the manuscript, and detailed responses are provided below.

**Comment 1:** First, in the outcomes, please specify that GFR slope was an outcome that was assessed in the study. Please also specify that the lab measurements, including proteinuria and creatinine, were collected after initiation of finerenone. This is implied, but is not explicitly stated in the outcomes.

**Response 1:** Thank you for this helpful suggestion. We have revised the Outcomes section to explicitly state that the eGFR slope was one of the study outcomes assessed. In addition, we clarified that laboratory measurements, including serum creatinine and proteinuria, were collected during follow-up after finerenone initiation and were used to evaluate changes over time. These revisions have been incorporated into the outcome section.

**Comment 2**: How are comorbidities defined? (ex. HF, CVD, etc)

**Response 2:** Thank you for this comment. Comorbidities, including heart failure (HF), cardiovascular disease (CVD), and other baseline conditions, were identified using documented diagnoses in the electronic medical records and were defined according to the corresponding ICD-10 codes. We have clarified this in the Methods section of the revised manuscript.

**Comment 3**: The following statement is in the methods but instead should be in the results (see below). "The estimated eGFR slope after finerenone 169 initiation was −1.08 mL/min/1.73 m² per month (95% CI −1.74 to −0.41; P = 0.002), corresponding 170 to an annualized decline of approximately −12.9 mL/min/1.73 m² per year. This indicates a 171 statistically significant decline in kidney function over follow-up (Figure B). Age and baseline uPCR 172 were not significantly associated with eGFR trajectory.”

**Response 3:**

Thank you for this important comment. We agree that this statement describes study results rather than methodology. Accordingly, it has been removed from the Methods section and relocated to the Results section.

**Comment 4:**

In the results section the authors state that 89.6 % of the patients had diabetes. However if having diabetic kidney disease is a prerequisite for the study, shouldn't 100% of the patients have diabetes. Further, is this biopsy-proven or presumed diabetic kidney disease? Please clarify this in the methods section.

**Response 4:**

We would like to thank you for this important comment. We have revised the Methods section to clarify that DKD was not a prerequisite for all included patients. The cohort included patients who received finerenone in routine clinical care either for documented DKD based on KDIGO-aligned clinical criteria **and/or** a urine protein-to-creatinine ratio (uPCR) >0.3 mg/mg.

We also clarified that DKD diagnosis was based on KDIGO-aligned clinical criteria and was not biopsy-proven.

**Comment 5:**

The authors state that the slope of the eGFR is approximately -12 mL/min/1.73 m²/year. This is extraordinarily high and would suggest the on average participants are losing 36 mL/min/1.73 m² in 3 years. This slope is considerably higher than the values reported for other agents. Are there any previous data for GFR slope with Finereone and how does this value compare to previous published values? I suspect this value in inflated due to short follow up time (3 months) where the GFR value falls more steeply before stabilizing. Can the author expand on this? This can be expanded on the discussion.

**Response 5:**

Thank you for this important comment. We agree that the annualized eGFR slope reported in our study appears steeper than values reported in previous finerenone studies and should be interpreted cautiously. In our cohort, the estimate was derived from a relatively short follow-up period and was driven mainly by early post-initiation eGFR changes. Therefore, it may not reflect the chronic long-term eGFR trajectory. We have revised the Discussion to clarify that the −12.9 mL/min/1.73 m²/year value represents an early annualized estimate rather than sustained long-term kidney function loss. We also added a comparison with prior real-world finerenone data, where an early eGFR decline has been observed before a flatter chronic slope. Finally, we acknowledged that short follow-up and missing later eGFR readings may have inflated the annualized estimate.

**Comment 6:**

Figure B> Pe-tamement is a typo and I think the authors meant "pre-treatment"

**Response 6:**

Thank you for pointing this out. The typo has been corrected to “pre-treatment” in both figures and manuscript files.

**Comment 7:**

Please review your reference list to ensure that it is complete and correct. If you have cited papers that have been retracted, please include the rationale for doing so in the manuscript text, or remove these references and replace them with relevant current references. Any changes to the reference list should be mentioned in the rebuttal letter that accompanies your revised manuscript. If you need to cite a retracted article, indicate the article’s retracted status in the References list and also include a citation and full reference for the retraction notice.

**Response 7:**

We appreciate the editor's comment. The reference list was checked for accuracy, completeness, and publication status. All cited references were verified using the original sources, and no retracted articles were identified.
